# Correlating Gray Matter Volume with Individual Difference in the Flanker Interference Effect

**DOI:** 10.1371/journal.pone.0136877

**Published:** 2015-08-31

**Authors:** Changming Chen, Jiemin Yang, Jiayu Lai, Hong Li, Jiajin Yuan, Najam ul Hasan Abbasi

**Affiliations:** 1 School of Psychology, Southwest University, Chongqing, China; 2 Key Laboratory of Cognition and Personality (SWU), Ministry of Education, Chongqing, China; 3 Hope Primary School, Chengjiang Town, Beibei, Chongqing, China; 4 Research Center for Brain Function and Psychological Science, Shenzhen University, Guangdong, China; 5 Department of Education, Nanchang Normal University, Nanchang, Jiangxi, China; 6 Department of Psychology, University of Sindh, Jamshoro, Sindh, Pakistan; Liaoning Normal University, CHINA

## Abstract

The Eriksen Flanker task has been widely used as a measurement of cognitive control, however till now information is still scarce about how the neuroanatomical properties are related to performance in this task. Using voxel-based morphometry technique (VBM), the current study identified a set of distributed areas where the gray matter volume (GM) correlated positively with participants’ efficiency in interference inhibition. These areas included the bilateral prefrontal gyri, left insula and inferior temporal gyrus, the left inferior parietal lobule. Further analysis using a novel machine learning algorithm with balanced cross-validation procedure confirmed that in these areas the GM-behavioral association was unlikely a byproduct of outlier values, instead, the gray matter volume could predict reliably participants’ interference inhibition efficiency. These results underscore the importance of the fronto-parietal and insula systems to the brain functioning of interference inhibition from the neuroanatomical perspective.

## Introduction

Efficient task execution requires fast and accurate target orienting and processing. But quite frequently, the target isn’t presented alone but accompanied by some distracting stimuli irrelevant to our goal. Therefore, to optimize performance we have to filter out surrounding irrelevant information and suppress its interference. Cognitive control therefore is a crucial function during the up-dating of goals and intentions [1], and to explore its neural substrates constitutes a fundamental mission for both the psychology and cognitive neuroscience-communities.

Among the various experimental paradigms measuring cognitive control, the Eriksen Flanker task is one of the top choices (see reviews [1, 2]). In this task, participants are asked to identify a central target stimulus such as an arrow, while ignoring distractors at the flanker positions. In the incongruent trials the flankers are associated with a response conflicting with the target stimulus, while in the congruent trials response in the flanker positions is just the same as the target. Therefore, the incongruent trials involve response conflict between the target and distractors, whereas the congruent trials do not. In some studies [3, 4] a third type of trials—the neutral trials—was also used, in these trials the flanker stimuli are physically distinguishable from the target stimuli but don’t associate with any conflicts or facilitation. Typically, participants show lower accuracy and longer reaction time in the incongruent condition than the other two, especially the congruent condition. This difference, referred as the flanker interference effect, is widely used as a measure of detection and resolution of response conflict [5].

Over the years, fMRI studies have been conducted exploring the neuro-functional mechanism underlying this effect [1],and these studies have revealed higher BOLD activation in the incongruent relative to the congruent or neutral conditions across several cortical regions, including the frontal system (the inferior frontal gyrus/VLPFC, middle frontal gyrus/DLPFC, superior frontal gyrus), the parietal system (inferior parietal lobule, superior parietal lobule), bilateral insula, as well as other regions like precentral gyrus, SMA and inferior temporal cortices [1, 4, 6–24]. In a review article in 2007, Nee and colleagues found consistent involvement of right DLPFC and right insula in the Eriksen flanker task[2]. A recent meta-analysis on 19 experiments identified significant convergence of activity in a bilateral dorsal fronto-parietal network consisting of dorsal premotor cortex and the superior parietal cortices, as well as activities in right inferior frontal junction and adjacent inferior frontal gyrus (pars opercularis), right anterior insula and the anterior midcingulate cortex extending into pre-supplementary motor area behind the Flanker interference effect [1]. Activities in these areas have been found to be closely associated with individual difference in interference inhibition function [3, 9, 22], and are clinically meaningful for distinguishing individuals with difficulties in cognitive control, such as the obsessive-compulsive disorder [14].

Using techniques such as Voxel-Based Morphometry (VBM), studies in the past decades have documented close linkage between brain anatomical properties and the individual difference in cognitive function (see a review: [25]). To our knowledge, till now only two articles have been published addressing the neuroanatomical relevance of the flanker interference effect and both are focusing on elderly people. When comparing good and poor performers in the Eriksen flanker task, Colcombe and colleagues [9] found that the good-performing elderlies showed significantly greater concentration of gray matter than poor performers in a left-lateralized portion of the anterior superior frontal gyrus, but showed no difference in the middle frontal gyrus, ACC and SMA. In a later study, Luks and colleagues [26] explored the relationship between regional brain atrophy and the performance of neurodegenerative patients. They found that the deterioration in interference inhibition was associated with atrophy in the lateral prefrontal cortex bilaterally, DLPFC, extending to the frontal pole and orbitofrontal cortex, right VLPFC and right temporal-parietal junction, as well as left anterior cingulate cortex and cerebellum.

Since these two studies were conducted on elderly or patients[26, 27], questions remain wide open if their findings could be generalized to healthy young adults, because aging could alter the brain structure and function of cognitive control [26, 27]. However, up to now no reports are available about how flanker interference effect is associated with the brain structural properties in young healthy adults. In light of this, the current study was decided to explore the relationship between brain morphometry with variability in interference function of normal people. We estimated the gray matter volume from high-resolution brain structural images of college students using the VBM technique, and explored regions that would show significant association with the flanker interference effect by voxel-wise general linear model. We also conducted a novel validation analysis using machine learning algorithms with a balanced threefold cross-validation procedure [28, 29] to verify the robustness of the neural-behavioral correlation in the areas identified

## Materials and Methods

### Ethics Statement

The current study was approved by the Human Research Ethics Committee of the Southwest University of China. Consents were obtained in written form from all the participants before their participation.

### Participants

Forty-two native Chinese students (30 females, 21.17±1.40 years old) at Southwest University, China were paid for participation. Two other participants were also recruited, but excluded for further analysis because of the low quality of structural images. All the participants were right-handed, with normal or corrected-to-normal visual ability. None of them had a history of neurology or psychiatric illness.

### Behavioral Test

After the MRI data acquisition, participants completed the arrow-version of Eriksen Flanker task. They were asked to judge the orientation of the central arrow, which was flanked by two stimuli on each side. The stimuli were presented with the E-Prime software (Psychological Software Tools, Pittsburg, PA, USA). The flankers were either arrows pointing to the same direction (congruent trials, e.g. >>>>>), or arrows pointing to the opposite direction as the central arrow (incongruent trials, e.g., >><>>), or asterisks (neutral trials, **<**). These three types of trials were interspersed across one session with 120 trials for each type. In each trial, the arrow image was presented for 500ms, then followed by a fixation jittering randomly from 1500ms to 3500ms. Trials from different conditions showed up in a pseudo-random way that there were no more than 3 consecutive trials with the same response, and no more than 3 consecutive trials from the same condition.

### Anatomical MRI Data Acquisition

Whole-brain high-resolution anatomical images were collected using a 12-channel head coil on a 3.0-T Siemens Trio MRI scanner (Siemens Medical, Erlangen, Germany) with a magnetization-prepared rapid gradient echo (MPRAGE) sequence. Parameters for the acquisition were as below: TR/TE/TI = 1900ms/2.52ms/900ms, flip angle = 9°, number of slices = 176, slice thickness = 1.0 mm, matrix = 256 × 256, voxel size = 1 × 1 × 1 mm^3^.

### Behavioral Data Analysis

For each participant, trials met any of the following criteria were discarded: 1) no response, 2) reaction time less than 200ms or more than 2000ms, or beyond 3 standardized variations away from the individual mean. As a result, 8.1% of the total trials were excluded. Mean accuracy and reaction time in each condition were calculated. Repeated measurement one-way ANOVA analysis was conducted to compare the accuracy and reaction time across three conditions.

### VBM Analysis

Optimized voxel-based morphometry analysis was conducted using the VBM8 toolbox (C. Gaser, Department of Psychiatry, University of Jena, Germany; http://dbm.neuro.uni-jena.de/vbm8) in Statistical Parametric Mapping 8 (SPM8; Wellcome Trust Centre for Neuroimaging, University College London, England). Firstly, scanner artifacts and gross anatomical abnormalities in T1-weighed images were checked, preprocessing was then carried out. Specifically, the images were corrected for bias field inhomogeneities, spatially normalized, segmented into GM, WM and cerebrospinal fluid (CSF) within the same generative model [30]. The segmentation procedure was further modulated by accounting for partial volume effects, applying adaptive maximum a posteriori estimations without the need for a priori information of tissue probabilities, and using a hidden Markov random field model [31]. The outputs of segmentations were inspected visually to monitor the quality. Finally, GM images were smoothed with a Gaussian kernel of 10 mm full width at half maximum. The kernel size was decided based on the findings in a simulated study by Shen and Sterr [32], as well as the recommendation by Christian Gaser and colleagues (http://www.neuro.uni-jena.de/vbm/segmentation/modulation/, see also in [33]),

### Behavior-VBM Correlation Analysis

Voxels-wise multiple regression was carried out to examine the relationship between GM volume and interference inhibition, with gray matter volume as the dependent variable, the indices of interference control as predictors of interests, and gender and age as variables of no interest. Given that the participants varied in their overall response accuracy and speed, we used the normalized indices of reaction time cost and accuracy cost as the predictors of interests, which were estimated as follow: normalized accuracy cost = (incongruent_acc-congruent_acc) /neural_acc, normalized reaction time cost = (incongruent_rt-congruent_rt)/neutral_rt. To avoid possible edge effects between different tissue types, we masked out all voxels with GM values less than 0.20 (absolute threshold masking). Clusters were deemed significant by applying a voxel-wise threshold of p<0.005 with a minimum of 470 contiguous voxels, corrected for multiple comparison within this gray matter mask using the ‘3dClustSim’ in AFNI program [34] based on Monte-Carlo simulation.[34]

### Results Validation

Since the conventional regression models assess correlation coefficients, which are sensitive to outliers and likely correlational with no predictive value [29, 33, 35], we used a machine learning algorithm with balanced cross-validation [28, 29] to confirm the robustness of the relation between interference inhibition and GM volume in—clusters identified above (see S1 Script for the Matlab codes). For each cluster, we estimated the mean GM volume in each region by Marsbar [36], then divided the GM volume and behavioral data for all participants into 3 folds in a balanced way. Two folds were used as the training set to build a linear model between GM volume and interference inhibition by regression, with normalized reaction time cost as the dependent variable, regional GM volume as the variable of interest while controlling gender and age. The model was then used to estimate the expected reaction time cost based on GM data in the remaining fold (the predicting set). The predicted values were then correlated with the observed reaction time cost in the remaining fold to reflect the fitness of the model. This procedure was repeated recursively that each fold was used as the predicting set once, and a mean *r*(expected, observed) was obtained based on these three iterations. This cross-validation process was iterated for 1000 times to get the final mean *r*(expected, observed). To assess the robustness of the contribution of regional gray matter volume, we also conducted similar 1000 iterations by randomly permuting the regional GM volume across participants, then contrasted the resulted distribution of *r*(expected, observed) with the final mean *r*(expected, observed) based on real data. It was hypothesized that if the regional GM volume was predictive for the efficiency of interference inhibition, randomly reallocating this variable would reduce the predictability of the model. Therefore, significance of the model was concluded if the mean correlation based on the real data was higher than 95% of the correlation coefficients based on the permuted data.

## Results

### 1 Behavioral Results

**Fig 1:**
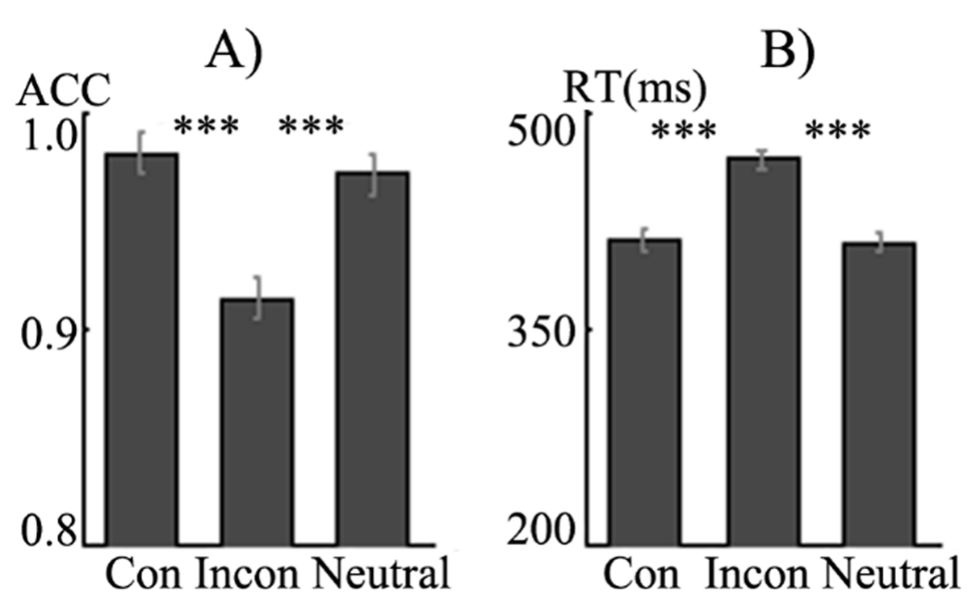
Accuracy and reaction time in the conditions.

Analysis on accuracy among the three conditions (Fig 1) revealed a significant main effect of condition (*F*(2,82) = 78.03, *p* = 0.00). Accuracy in the congruent trials was significantly higher than that in the incongruent (*t*(41) = 10.06, *p* = 0.00) and neutral trials (*t*(41) = 3.76, *p* = 0.00), meanwhile, accuracy in the neutral condition was higher than that in the incongruent condition (*t*(41) = 8.15, *p* = 0.00). As for reaction time, there was also a significant main effect of condition (*F*(2,82) = 385.15, *p* = 0.00), participants responded significantly slower in the incongruent than the congruent (*t*(41) = 21.26, *p* = 0.00) and neutral conditions (*t*(41) = 19.90, *p* = 0.00), but equally fast between the congruent and neutral conditions (*t*(43) = -1.57, *p*>0.05).

### 2 VBM results

#### 2.1 Behavior-VBM correlation

**Fig 2:**
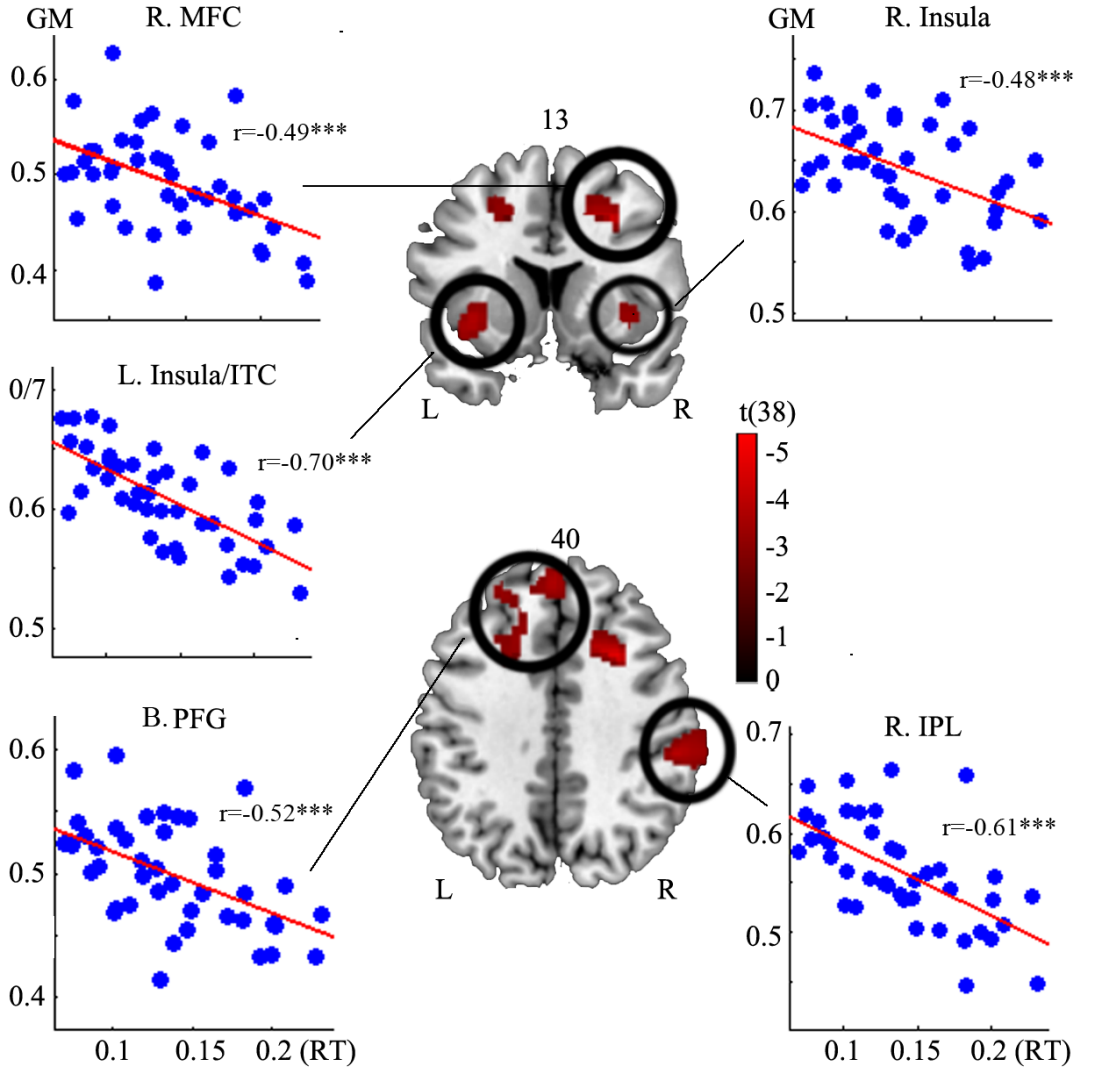
The clusters revealed by the voxel-wise linear regression model. In these clusters, the gray matter volume (vertical axis) correlated negatively with the normalized reaction time cost (horizontal axis, calculated as follow: (rt_incongruent-rt_congruent)/rt_neutral.

The multiple regression analysis revealed five major clusters with significant negative correlation between gray matter volume and the normalized reaction time cost (height threshold: *p*<0.005, extent threshold: 470 voxels, corrected for multiple comparisons), encompassing the left insula/superior temporal gyri/left inferior temporal/parahippocampal gyri, bilateral prefrontal gyri (extending from the left superior frontal gyrus, left medial frontal gyrus to the right superior frontal gyrus and medial frontal gyrus), right inferior parietal lobule, right middle frontal gyrus (Fig 2 and Table 1). No clusters were found demonstrating positive correlation with the normalized reaction time cost. No clusters were found to show significant correlation between regional gray matter volume and normalized accuracy cost either.

**Table 1:** Clusters correlating with interference inhibition.

Region	X	Y	Z	Peak t	BA	No. voxels	*p*(cross-validation)
B. Prefrontal Gyri	-14	60	31	-3.88	6/8/9/10	2934	0
L. Insula/ITG/PPG	-39	3	-9	-5.36	13/34/28/21	7458	0
R. Insula	38	5	-5	-3.83	13/34	1602	0.007
R. Mid. Frontal Gyrus	29	14	37	-5.57	8	1757	0
R. Inf. Parietal Lobule	57	-31	34	-5.19	40	2312	0

Since a large number of previous studies on flanker effect used unnormalized indices of interference inhibition (e.g. unnormalized reaction time cost and unnormalized accuracy cost, [22, 26]), we also conducted multiple regression analysis using the unnormalized indices as predictors of interests to see if the results would converge with those based on normalized indices. We observed a very high positive correlation between the normalized and unnormalized reaction time cost(*r*(42) = 0.949, *p* = 0.00*)*, as well as the normalized and unnormalized accuracy cost (*r*(42) = 0.99985, *p* = 0.00*)*. Regions identified in this step were also similar to those observed when normalized indices were used (see supplementary information).

### 2.2 Validation Results

**Fig 3:**
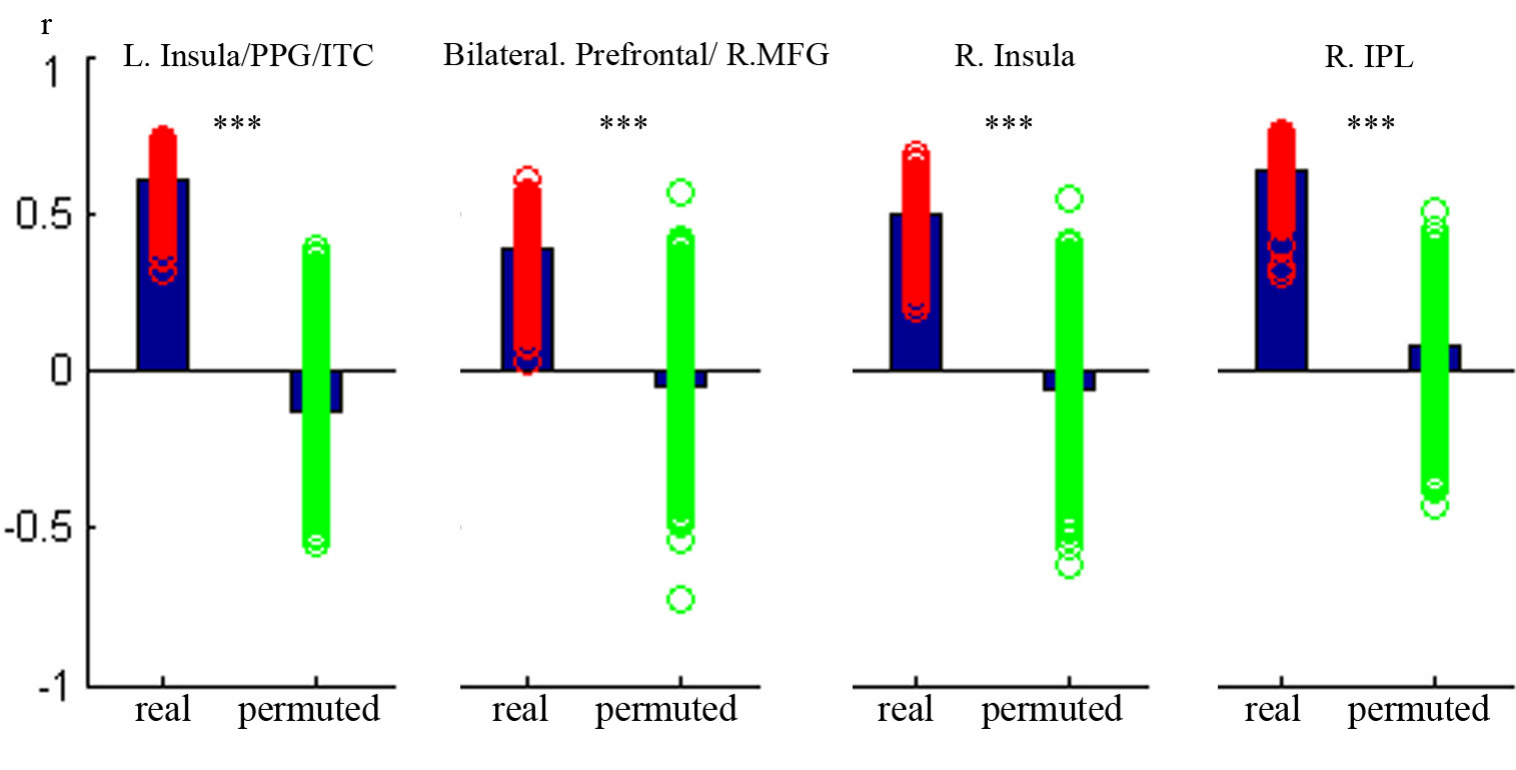
Results in the three-fold balanced cross-validation analysis. To assess the significance of the contribution of interference inhibition, we contrasted the mean *r*(predicted, observed) in the real data (the left bar) with the distribution of *r*(predicted, observed) when the normalized reaction time cost was permuted (the right bar). Significance was concluded if the final mean *r*(predicted, observed) based on the real data was higher than 95% of the correlation coefficients estimated from the permuted data. *Height of the left bar*: mean correlation in the 1000 iterations based on real data. *Scatter around the left bar*: distribution of correlation coefficient in the 1000 iterations based on real data. *Height of the right bar*: mean correlation based on permuted reaction time cost. *Scatter around the left bar*: distribution of correlation coefficients in the 1000 iterations based on permuted data. *Vertical axis*: *r*(observed, predicted).

To explore the robustness of the behavioral-neural relationship especially if these clusters identified above were byproducts of outlier values, we conducted additional validation analysis using machine learning algorithms with a balanced threefold cross-validation procedure. The results showed that in all the five clusters, the final mean *r*(observed, predicted) based on originally unpermuted data (real data) was within the top 1% of the *r*(observed,predicted) based on simulated random data (Fig 3), which suggested that outlier values were unlikely driving the GM-behavioral correlation in these clusters.

## Discussion

Using VBM technique, we identified in this study a set of distributed regions where the gray matter volume associated closely with participants’ efficiency in interference inhibition. These regions included the bilateral prefrontal gyri, right middle frontal gyrus, bilateral insula and inferior temporal cortex, as well as the left inferior parietal lobule. The GM-behavioral relationship in these clusters was strengthened by following-up validation analysis, where we found a significant decrease in the predictability of regional gray matter volume to the efficiency in interference inhibition once the gray matter values were randomly permuted across participants. These results suggested that the GM-behavioral association was unlikely due to outlier values, but rather stable.

The clusters found in the current study dovetailed with the results in previous fMRI studies. For instance, Casey and colleagues [8] proposed four distinct neural subsystems which seemed to be involved differentially in the performance of the flanker task, including the anterior system involving the anterior cingulate cortex and dorsolateral prefrontal cortex, a visuospatial attention system involving the right superior frontal gyrus, superior parietal cortex, and portions of the right cerebellum, a third network involving the caudate nucleus and insula, and a fourth system involving the inferior parietal region and also portions of auditory cortex. They were also consistent with the results in previous meta-analysis which pinpointed the contribution of DLPFC, insula, parietal cortex to the Flanker interference effect [1, 2].

Cognitive neuroscience has witnessed a plethora of theoretical accounts for the neural mechanism of cognitive control. As one prominent view, Dosenbach, Petersen and colleagues [37, 38] divided the nodes of the control system into two major networks according to their functional properties and connectivity: the ‘fronto-parietal’ and ‘cingulo-opercular’ networks. The fronto-parietal network consists of the dlPFC (middle frontal gyrus), inferior parietal lobule (IPL), dorsal frontal cortex (dFC), the intraparietal sulcus (IPS), precuneus and middle cingulate cortex (mCC). It is thought to initiate and adjust control and act as a flexible hub for cognitive control [39, 40]. This network shows predominantly feedback and adjustment (dlPFC and IPL), or control initiation activity (IPS and dFC), and can alter its functional connectivity with other networks according to task demands [39, 40]. The cingulo-opercular network includes the anterior prefrontal cortex (aPFC), anterior insula/ frontal operculum (aI/fO), dACC/msFC and thalamus, which carries task set-maintenance signals and provide stable ‘set-maintenance’ over entire task epochs.

As a core component of the fronto-parietal network, the middle frontal gyrus (or the dorsolateral prefrontal cortex, DLPFC) has been found involved in a range of inhibition tasks, such as the Go/No-go task [41–43], see a meta-analysis, [44]; stroop task ([45, 46] see the review: [2]). Activity in this region has been proposed to associate with control implementation and top-down modulation of attentional processes [47]. It has also been thought to implement the attention controller subsystem that exerts cognitive control, and play a pivotal role in the dynamic tuning of executive control during any kind of conflict-induced behavioral adjustments [48]. In the Eriksen Flanker task, the middle frontal gyrus (MFG) has been consistently found to elicit significantly higher response to the incongruent trials than to the congruent or neural trials in fMRI studies [3, 4, 6, 8, 9, 12, 15, 17, 19, 21–23]).This has been observed regardless of the stimuli materials, no matter they are letters, color squares [4], or arrows. The congruency effect in this area has been found to be associated closely with participants’ efficiency in interference inhibition [3]. For instance, Bunge[3] reported that in MFG the activation difference between incongruent with neutral conditions correlated negatively with the reaction time difference between these two conditions, and an study by Colcombe [9] on elderly adults found the good performers in the Eriksen Flanker task demonstrated significantly higher activation than the poor performers in the incongruent condition. From the neuroanatomical perspective, the study on elderly patients with neurodegenerative disease by Luks [26] observed positive correlation between interference susceptibility and atrophy in DLPFC. Several hypotheses have been proposed about the detailed role of MFG behind the Flanker interference effect. Nee [2] proposes that right MFG (DLPFC) is involved in interference resolution during response selection, but less likely due to stimuli conflict or response selection conflict during the Flanker task. [23] suggests that the prefrontal activation might not be specific for error processing but for the implementation of control (i.e., task set management) when either response conflict or errors are detected. Blasi [6] found that the MFG was more activated during response inhibition than during interference monitoring, which they thought could add evidence to the view that DLPFC was particularly relevant for the top-down modulation of the processing of cognitive stimuli, for control implementation and for building representations that bias behavior towards an appropriate response or set of features [47, 49].

In conjunction with the importance of MFG, the current study also observed positive relationship between interference inhibition function with gray matter volume in inferior parietal lobule. Given its close link to control of spatial attention [50–52], in inhibition tasks like the Eriksen Flanker task, this region may monitor conflicts between representations of visual inputs and signal lateral prefrontal cortex to conduct control [53]. In inhibition tasks, this region was suggested to be related to the storage of stimuli-response representation rather than inhibition [54]. Casey [8] suggested that the inferior parietal region may be more involved in broadening of attention beyond the fovea to include the periphery. Activity in this region might also be attributed to sudden and robust disengagement of attention from its current focus [47]. The association between inferior parietal lobule and flanker interference effect observed in this study is consistent with previous fMRI studies comparing BOLD response in the incongruent and congruent trials [3, 4, 6, 12, 13, 15–18, 20, 23, 24]. In these studies, the incongruent trials were found to elicit significant response than the congruent trials, and like the DLPFC and superior frontal gyrus, congruency effect in this region were observed regardless of the stimulus materials [4].

In fMRI studies, activity in the parietal cortex has been attributed partly to its connectivity to prefrontal cortex. In the current study, we also observed positive association between interference inhibition efficiency and gray matter volume in the superior frontal gyrus (SFG), which was consistent with findings in the two VBM studies by Colcombe et al [9] and Luks et al [26]. In Colcombe’s study [9], the good elderly performers of the Eriksen Flanker task demonstrated higher gray matter concentration than their poor counterparts in anterior SFG, whereas in Luks’ study [26] the magnitude of interference susceptibility (indexed by accuracy difference between incongruent and congruent conditions) was found to correlate positively with decrease in gray matter in this region. Previous studies on cognitive control have suggested that the superior frontal gyrus is critical for monitoring and adjusting downstream cognitive processors [37, 55], it associates with a network controlling goal-directed behavioral through the stable maintenance of task sets, and is thought to enable top-down control by biasing processing in other brain regions toward contextually appropriate representations [37, 56]. In a number of fMRI studies on the Eriksen Flanker task, this region has been found to demonstrate higher BOLD activation to the incongruent condition than the congruent or neutral condition [3, 4, 6, 8, 10–12, 16, 19, 22, 24]. Activation in this region during the Flanker task has been thought to fit with the role of the visuospatial attention system in orienting of attention to the relevant target location and guiding the eye to an appropriate area of the visual field [8].

Another important area identified located in bilateral insula. This area is associated with the subjective awareness, erroneous actions [57], and subserves cognitive functions like saliency, task switching, attention and control (see reviews: [58, 59]). It has been suggested to provide a link between attention-related problem solving and salience systems regardless of perceptual domain (auditory or visual) or mode of response (word production or button press) during the coordination and evaluation of task performance [18]. In the dual model of cognitive control [37] the insula is thought to play crucial role in providing stable ‘set-maintenance’ over entire task epochs. In previous studies, this area demonstrates higher response to incongruent trials over the congruent or neutral trials in the Eriksen Flanker task [3, 6, 10–12, 14–19, 21–23, 60]. The insula is one of the most stable areas where the flanker congruency effect has been observed (see reviews: [1, 2]). In this region, the neural congruency effect has been found correlated negatively with the efficiency of interference inhibition based on reaction time difference[3]. Activation in this region has been found to increase when the congruency of the trial contradicts the preceding trials, which suggests it is also sensitive to violations in expectancy or sudden changes in the frequency of an event [8].

In summary, our results underscored the importance of regions in the fronto-parietal and insula systems to the flanker interference effect, by providing novel findings from the neuroanatomical perspective about their close association with participants’ efficiency in interference inhibition. In light of the methodological and theoretical importance of Eriksen Flanker Task, these results could enrich the information and contribute to our understanding of the neural mechanism of cognitive control.

## Supporting Information

S1 Fig The clusters correlating negatively with unnormalized reaction time cost. The horizontal axis in scatter plot represents the unnormalized reaction time cost, calculated by subtracting the reaction time in the congruency trials from that in the incongruent trials. The vertical axis represents the regional mean gray matter volume.
(TIF)

S2 Fig The validation result in each cluster. (TIF)

S1 Script kfold_crossvalid_bymultipleregression.m (M)

S1 Text Supplementary results. (DOC)
