# Supplementary figures and images for: Correlating Gray Matter Volume with Individual Difference in the Flanker Interference Effect
Source: PLoS One. 2015 Aug 31;10(8):e0136877. doi: 10.1371/journal.pone.0136877 (PMC4554993; doi:10.1371/journal.pone.0136877)

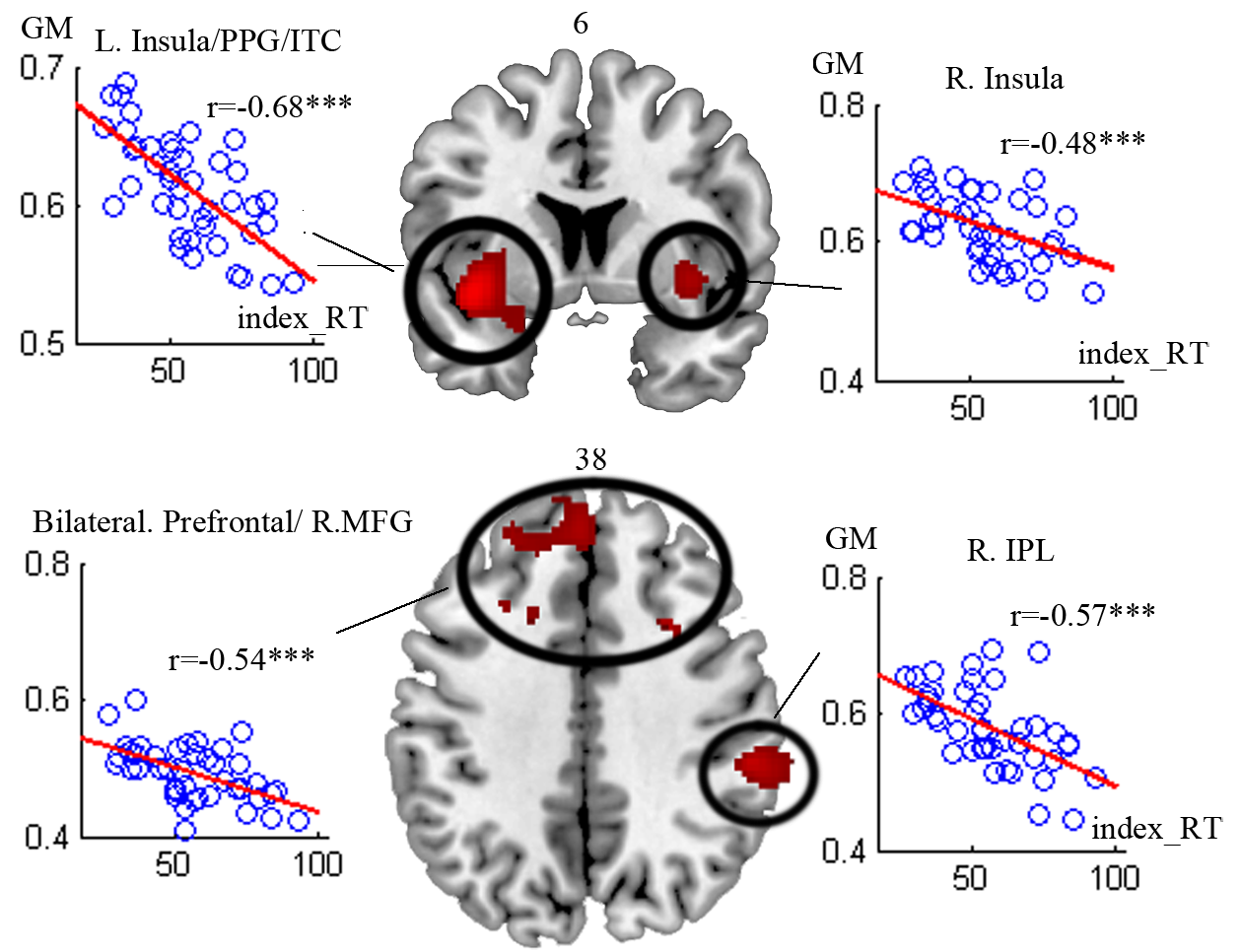

Supplement: S1 Fig — The horizontal axis in scatter plot represents the unnormalized reaction time cost, calculated by subtracting the reaction time in the congruency trials from that in the incongruent trials. The vertical axis represents the regional mean gray matter volume. (TIF) [file pone.0136877.s001.tif]

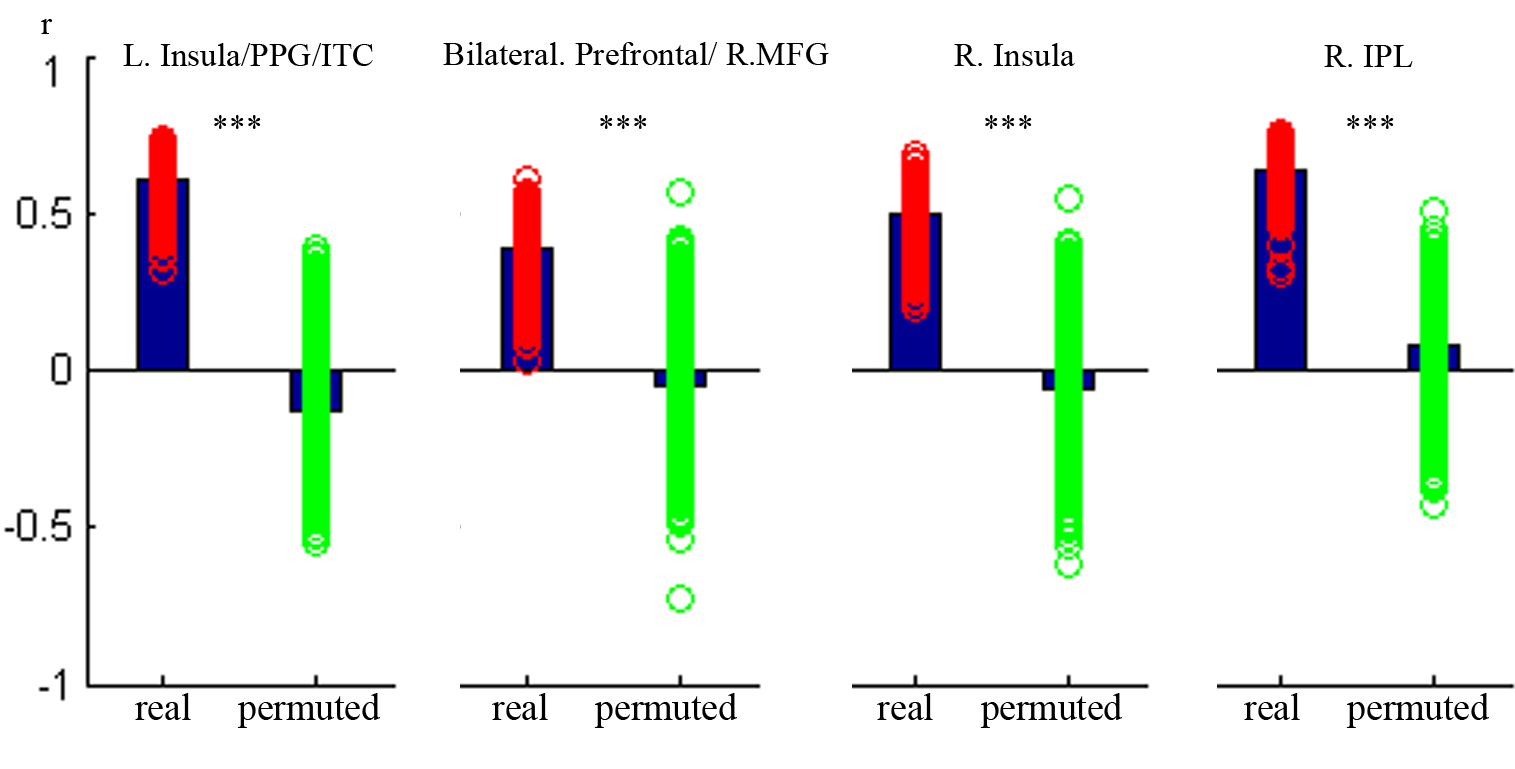

Supplement: S2 Fig — (TIF) [file pone.0136877.s002.tif]
