# Supplementary material for: Correlating Gray Matter Volume with Individual Difference in the Flanker Interference Effect
Source: PLoS One. 2015 Aug 31;10(8):e0136877. doi: 10.1371/journal.pone.0136877 (PMC4554993; doi:10.1371/journal.pone.0136877)
Supplement: S1 Text — (DOC) [file pone.0136877.s004.doc]

**Text S1. Supplementary results.**

Correlating Gray Matter Volume with Individual Difference in the Flanker Interference Effect—Supplementary Results

Given that a large number of behavioral or functional MRI studies on Eriksen Flanker task (e.g. ) used the unnormalized index of accuracy cost (acc_incongruent-accc_congruent) and reaction time cost (rt_incongruent-rt_congruent), we also explored the behavioral-neural correlation using the unnormalized indices, especially to see if the results would converge with those observed when normalized indices were used.

First, we estimated the Pearson correlation between the normalized and unnormalized indices. We found a very high correlation between the unnormalized and normalized reaction time cost, r(42)=0.949, p=0.000, as well as a high correlation between the unnormalized and normalized accuracy cost, r(42)=0.99985, p=0.000. There was no correlation between the unnormalized accuracy cost and unnormalized reaction time cost, r(42)=.134, p>0.05, nor correlation between the normalized accuracy cost and normalized reaction time cost, r(44)=0.185, p>0.05.

Next, multiple regression analysis was conducted as in the main text, which revealed four major clusters whose gray matter volume correlated negatively with the unnormalized reaction time cost (corrected for multiple comparisons, see **Table S1** and **Fig. S1**), and the locations for these clusters overlapped largely with those based on normalized reaction time cost. The first cluster extended from the left insula, superior temporal gyrus, parahippocampal gyrus to left inferior temporal gyrus, the second cluster located within the bilateral superior, medial and medial frontal gyri. The third cluster covered mainly the right inferior parietal lobule, and the fourth one covered the right insula.

The validation analysis confirmed the predictability of unnormalized reaction time cost to gray matter volume in the regions identified above. In all these four clusters, the final mean r(predicted, observed) in originally unpermuted data was within the top 1% of the distribution of r(predicted, observed) estimated when the unnormalized reaction time cost was randomly permuted (p<=0.005, see **Fig.** **S1** and **Fig. S2**).

------------

Table S1. Clusters Correlating Negatively with Unnormalized Reaction Time Cost

| Region | X | Y | Z | Peak t | BA | No. voxels | P(cross-validation) |
| --- | --- | --- | --- | --- | --- | --- | --- |
| L. Insula/ITG/PPG | -39 | 5 | -9 | -5.10 | 13/34/28/31 | 7131 | 0.000 |
| R. Insula | 38 | 5 | -5 | -4.13 | 13/34 | 1033 | 0.001 |
| Bilateral Prefrontal Gyrus/ R.MFG | 29 | 14 | 37 | -4.35 | 6/8/9/10 | 4072 | 0.000 |
| R. Inf. Parietal Lobule | 56 | -34 | 33 | -4.55 | 40 | 1348 | 0.000 |

(Height threshold: p<0.005, extent threshold: 470 contiguous voxels)

--------

References:

Bunge, S. A., Hazeltine, E., Scanlon, M. D., Rosen, A. C., & Gabrieli, J. D. (2002). Dissociable contributions of prefrontal and parietal cortices to response selection. *NeuroImage, 17*(3), 1562-1571.

Luks, T. L., Oliveira, M., Possin, K. L., Bird, A., Miller, B. L., Weiner, M. W., & Kramer, J. H. (2010). Atrophy in two attention networks is associated with performance on a Flanker task in neurodegenerative disease. *Neuropsychologia, 48*(1), 165-170.

Ochsner, K. N., Hughes, B., Robertson, E. R., Cooper, J. C., & Gabrieli, J. D. (2009). Neural systems supporting the control of affective and cognitive conflicts. *Journal of cognitive neuroscience, 21*(9), 1842-1855.
